# Analysis of Sexual Risk Behaviors and Psychosocial Determinants Among Older MSM in Mainland China and Hong Kong

**DOI:** 10.1093/geroni/igaf122.3705

**Published:** 2025-12-31

**Authors:** Alex Siu Wing Chan, Hok Bun Ku, Elsie Yan

**Affiliations:** Hong Kong Baptist University, Hong Kong, China (People’s Republic); The Hong Kong Polytechnic University, Hong Kong, China (People’s Republic); The Hong Kong Polytechnic University, Hong Kong, China (People’s Republic)

## Abstract

Older men who have sex with men (OMSM) in Asia face significant sexual health and mental health challenges, yet they are often overlooked in HIV prevention efforts. The psychosocial factors, including substance use, mental health issues, and social stigma, further exacerbate these risks. This study aims to investigate the sexual risk behaviors and psychosocial factors influencing older MSM in Mainland China and Hong Kong, with a focus on identifying key predictors of HIV risk. This cross-sectional comparative study surveyed 302 older MSM (151 from Mainland China and 151 from Hong Kong). Participants, aged 60 and above, were recruited through partnerships with LGBT organizations and NGOs. Data were collected on demographic characteristics, sexual risk behaviors, and psychosocial factors, including mental health and substance use. The study found that only 24.3% of participants aged 50–59 years and 17.1% of those aged 60–69 years reported using condoms at last intercourse. Furthermore, 25% of participants reported having multiple sexual partners. Substance use, particularly methamphetamine, was linked to decreased condom use, with 30% of participants reporting recreational drug use. Mental health issues, including depression and anxiety, were prevalent, with 40% reporting symptoms of depression. Social stigma was a significant barrier to seeking care, further exacerbating these issues. Older MSM in Mainland China and Hong Kong engage in high-risk sexual behaviors influenced by mental health struggles, substance use, and stigma. Tailored, culturally sensitive interventions that address both sexual health and mental health are urgently needed to reduce HIV and STI transmission in this underserved population.

